# Cost-Effectiveness of Dapagliflozin in the Treatment of Patients With Chronic Kidney Disease in Denmark

**DOI:** 10.1016/j.xkme.2026.101497

**Published:** 2026-08-12

**Authors:** Thea Tanderup Kjær, Morten Lindhardt, Anders Hammerich Riis, Henrik Birn, Lars Holger Ehlers

**Affiliations:** 1 Nordic Institute of Health Economics, Aarhus, Denmark; 2 Department of Clinical Medicine, University of Copenhagen, Copenhagen, Denmark; 3 Department of Medicine 1, Copenhagen University, Hospital Holbaek, Holbaek, Denmark; 4 Enversion A/S, Aarhus, Denmark; 5 Department of Clinical Medicine and Renal Medicine, Aarhus University and Aarhus University Hospital, Aarhus, Denmark

**Keywords:** Albuminuria, chronic kidney disease, costs and cost analysis, disease progression, sodium/glucose transporter 2 inhibitors

## Abstract

**Rationale & Objective:**

Chronic kidney disease (CKD) is a growing burden worldwide and in Denmark, with substantial clinical and economic consequences. Although national guidelines recommend sodium/glucose transporter 2 inhibitors (SGLT2i) for patients with CKD, type 2 diabetes (T2D) or heart failure (HF), underdiagnosis limits the use of SGLT2i. This study aims to inform decision makers in health care about the cost-effectiveness of current guidelines for CKD in Denmark focusing on SGLT2i dapagliflozin added to standard of care (SoC) versus SoC alone in patients with CKD.

**Study Design:**

Cost-effectiveness analysis based on a Markov model with a lifetime horizon.

**Setting & Participants:**

Danish health care payer perspective including a broad population with CKD and subgroups stratified by urinary albumin-creatine ratio (UACR), T2D status, and HF history.

**Exposures:**

Dapagliflozin plus SoC versus SoC alone.

**Outcomes:**

Quality-adjusted life years (QALYs), life-years, total costs, and incremental cost-effectiveness ratios (ICERs).

**Analytical Approach:**

A published Markov model was populated using data from the DAPA-CKD and DECLARE-TIMI 58 trials. Analyses were conducted for the overall population and stratified subgroups. Results were evaluated against an assumed willingness-to-pay (WTP) threshold of €24,158 per QALY.

**Results:**

Dapagliflozin treatment resulted in health benefits (QALYs) and life-years versus SoC irrespective of T2D/HF status or UACR levels. Dapagliflozin was cost-effective for the total population with an ICER of €7,499/QALY and dominant for subgroups with high UACR (≥200 mg/g) regardless of T2D/HF status. ICERs were below WTP threshold for all subgroups. However, for patients with CKD and low UACR (<200 mg/g) without T2D/HF, time to cost-effectiveness was long, and estimates were uncertain.

**Limitations:**

Results are limited by generalizability, long-term uncertainty, simulated subgroup data, cost uncertainty, and lack of a Danish WTP threshold.

**Conclusions:**

This study indicates that dapagliflozin added to SoC is cost-effective across CKD subgroups as recommended in the current Danish guideline recommendations.

## Plain-Language Summary

Chronic kidney disease (CKD) is placing a growing burden on patients and the health care system. Effective treatments exist and are recommended in guidelines, but many patients are not diagnosed or treated early enough. In this study, we assessed whether adding dapagliflozin to standard care provides value for money by using a simulation model based on clinical trial data to compare costs and health outcomes with standard care alone. We found that adding dapagliflozin improves patients’ health and can reduce the need for more intensive and costly treatments over time. Benefits were observed across patient groups, particularly in those with more advanced diseases. Overall, better implementation of existing guidelines could improve patient outcomes while making more efficient use of health care resources.

The global prevalence of chronic kidney disease (CKD) is likely to increase in the future because of population growth, aging, and increased numbers of people with diabetes and hypertension.^1^, ^2^, ^3^, ^4^ This has major clinical and societal impact as even mild or moderate kidney disease is associated with an increased risk of cardiovascular disease, acute kidney injury, and premature death.^5^ In Denmark, approximately 9.5% of the adult population has CKD.^6^ The median age at the time of CKD in Denmark is 74 years (interquartile range [IQR], 65-80 years), and currently one in four incident CKD patients die within 5 years.^6^

Sodium/glucose transporter 2 inhibitors (SGLT2i) reduce the risk of kidney disease progression and cardiovascular disease in patients with CKD.^7^, ^8^, ^9^ The DAPA-CKD trial was the first trial to show the benefits of SGLT2i in patients with CKD irrespective of type 2 diabetes (T2D) status by reporting significantly lower risk of the primary endpoints (sustained decline in the estimated glomerular filtration rate [eGFR] of at least 50%, end stage kidney disease [ESKD], or death from kidney or cardiovascular causes) and death from any cause for dapagliflozin versus standard of care (SoC).^7^ Subsequently, empagliflozin has been shown to decrease the risk of CKD progression or death from cardiovascular causes compared with placebo in patients with CKD with or without T2D.^8^

The current treatment guidelines for CKD in Denmark include lifestyle changes, blood pressure control as well as angiotensin-converting enzyme (ACE) inhibitors or angiotensin receptor blockers (ARBs) in patients with CKD and albuminuria or diabetes.^10^ Patients with CKD with eGFR > 20 mL/min/1.73 m^2^ and a urinary albumin-creatine ratio (UACR) > 200 mg/g are currently recommended SGLT2i.^10^ Furthermore, SGLT2i should be considered for all patients with heart failure (HF) or T2D depending on UACR and eGFR level.^11^^,^^12^ Barriers to optimal CKD management include implementation of guideline directed medical therapy, lack of a diagnostic code for CKD in primary care as well as underutilization of UACR measurements in primary care affecting the identification of early stages of CKD.^13^^,^^14^ Ultimately, the barriers limit the proportion of patients with CKD in Denmark receiving SGLT2i. Data suggest that less than 40% of the eligible population in primary care is prescribed SGLT2i.^14^ This raises questions regarding the priority in clinical practice and cost-effectiveness of SGLT2i according to the current CKD guidelines in Denmark. However, no prior Danish-specific evaluation of SGLT2i cost-effectiveness has been published, which could guide implementation and reimbursement.

This study aimed to investigate the lifetime cost-effectiveness of the current Danish guideline implementation for CKD in relation to UACR levels, diabetes status or history of HF, with focus on dapagliflozin added to the SoC (ACE and ARB) compared with SoC alone from a Danish health care payer perspective.

## Materials and Methods

### Study design

A cost-utility analysis (CUA) was conducted from a Danish national health care perspective to evaluate the cost-effectiveness of SGLT2i focusing on dapagliflozin plus SoC compared with SoC over a lifetime (30 years) horizon. This analysis was based on a previously published Markov model adapted to a Danish setting with updated model inputs using Danish registry data to reflect local epidemiology, clinical practice, and costs (Fig S1).^15^ The model data were derived from the DAPA-CKD and DECLARE-TIMI 58 trials and follow the approach of McEwan et al. 2024.^7^^,^^15^^,^^16^ The primary outcomes of the model were net monetary benefit (NMB) and incremental cost-effectiveness ratio (ICER) with effectiveness measured in quality adjusted life years (QALY) and life years (LY) gained. The ICER is the traditional summary result from a cost-effectiveness analysis that represents the additional cost required to achieve an additional unit of health benefit when comparing one health care intervention to another.^17^ In NMB, the difference in effect between 2 options being evaluated is rescaled into monetary value by multiplying the incremental effectiveness with the WTP threshold and subtracting the incremental costs.^17^ Given the absence of an official willingness-to-pay threshold (WTP) in Denmark, a conservative threshold at €24,158/QALY was applied corresponding to the lower end of the range (£20,000 to £30,000/QALY, GBP/EUR exchange rate = 9.01) commonly cited in National Institute of Health and Care Excellence’s (NICE) practice.^18^ The NICE value is widely applied as a conservative estimate of a threshold value in health economic evaluations in primary care in Denmark.^19^ Discounting of both costs and health benefits was set at 3.5% per annum according to the Danish Finance Ministry requirements.^20^

### Patient population

The broad Danish CKD population in the analysis was defined as eGFR between 15 and 59 mL/min/1.73 m^2^, or those with an eGFR between 60 and 90 mL/min/1.73 m^2^ and a UACR > 30 mg/g. The proportion of patients with low UACR was assumed to be 75% and the remaining 25% with high UACR (>200 mg/g) and corresponds to the distribution in the DECLARE_CKD_ population.^15^^,^^16^ In the baseline model population, no patients had kidney failure (ie, no patients received kidney replacement therapy” (Tables 1 and S1-S2). Given the unavailability of a single dataset providing all required data for the broad CKD population, a pooled population from the DAPA-CKD trial and the DECLARE-TIMI 58 trial were applied. However, the DAPA-CKD trial only included patients with an high UACR (≥ 200 mg/g), and the DECLARE-TIMI 58 trial only included patients with risk factors for atherosclerotic cardiovascular disease, T2D, and a creatinine clearance ≥60 mL/min.^7^^,^^16^ Thus, only the subset of participants from the DECLARE-TIMI 58 trial with an eGFR <60 mL/min/1.73 m^2^ and/or albuminuria at baseline were included, referred to hereafter as the DECLARE_CKD_ population.^15^^,^^16^ The effects of dapagliflozin on patients with low UACR (<200 mg/g) and without T2D were simulated based on adjustment factors relative to patients with T2D from the DAPA-CKD trial and Poisson distribution.

**Table 1:** Baseline Characteristics of the Total Population Divided by UACR (low vs high).^a^

Patient Characteristics	High UACR–25%	Low UACR–75%
Demographic characteristics		
Age, y	62.2 (0.15)	64.6 (0.11)
Female	32.1% (0.01)	33.7% (0.01)
BMI, kg/m^2^	30.4 (0.08)	32.4 (0.09)
Race		
White	59.3% (0.01)	77.6% (0.01)
Black or African American	4.3% (0.00)	3.6% (0.00)
Asian^b^	29.0% (0.03)	15.4% (0.02)
Other	7.4% (0.00)	3.4% (0.00)
Smoker	38.9% (0.01)	14.8% (0.01)
Clinical characteristics		
CKD G1	11.0% (0.00)	37.7% (0.01)
CKD G2	20.3% (0.01)	37.4% (0.01)
CKD G3a	25.1% (0.01)	20.6% (0.01)
CKD G3b	32.8% (0.01)	3.8% (0.00)
CKD G4	10.8% (0.00)	0.4% (0.00)
CKD G5 (pre-RRT)	0.02% (0.00)	0.1% (0.00)
Dialysis	0.0% (0.00)	0.0% (0.00)
Transplant	0.0% (0.00)	0.0% (0.00)
UACR: 30-300 mg/g	12.8% (0.00)	82.3% (0.01)
UACR: ≥300 mg/g	87.2% (0.00)	0.0% (0.00)
Type 2 diabetes	76.2% (0.01)	73.9% (0.00)
Glomerulonephritis	16.4% (0.01)	6.5% (0.02)
ACE	36.2% (0.01)	49.1% (0.01)
ARB	59.7% (0.01)	36.3% (0.01)
MRA	5.3% (0.00)	5.0% (0.00)
Diuretic	43.1% (0.01)	44.9% (0.01)
Potassium, mEq/L	4.6 (0.01)	4.5 (0.01)
Systolic blood pressure, mm Hg	138.5 (0.23)	135.8 (0.24)
Hemoglobin, g/dL	13.1 (0.02)	13.8 (0.02)
Medical history		
Prior HF	11.6% (0.00)	11.9% (0.01)
Prior MI	12.9% (0.00)	22.5% (0.01)
Prior stroke	7.5% (0.00)	7.4% (0.00)

Abbreviations: ACE, angiotensin-converting enzyme; ARBs, angiotensin receptor blockers; BMI; body mass index; CKD, chronic kidney disease; HF, heart failure; MI, myocardial infarction; MRA, aldosterone receptor antagonists; RRT, renal replacement therapy; T2D, type 2 diabetes; UACR; urinary albumin-creatine ratio.

a Unless otherwise indicated, values for continuous variables are mean (SE); values for categorical variables represent counts (%).

b SE 10 % of mean value.

### Disease progression

Within a monthly cycle, patients with CKD could transit between CKD stages G1 to G5, renal replacement therapy (RRT), or death. RRT included dialysis or kidney transplants. The transition probabilities and risks of serious adverse events (SAEs) in the model were derived from DAPA-CKD, DECLARE-TIMI 58 and a systematic literature review on CKD modelling and are described in detail elsewhere.^7^^,^^15^, ^16^, ^17^ The SAEs included volume depletion, major hypoglycemic events, fractures, diabetic ketoacidosis, and amputation. Each year the patients treated with dapagliflozin were assigned a discontinuation rate of 0.0617.^7^ Furthermore, patients discontinue dapagliflozin after kidney transplantation or when initiating dialysis. The incidence of all-cause mortality was extrapolated using adjusted parametric survival equations (Weibull) and adjusted for all-cause mortality from Statistics Denmark.^15^^,^^18^^,^^19^

### Cost

Direct costs from a health care payer perspective were based on the best available literature, Diagnosis Related Group tariffs, official medicines prices, and the Danish Medicines Council’s unit costs (Table 2).^20^, ^21^, ^22^, ^23^, ^24^, ^25^, ^26^, ^27^, ^28^, ^29^, ^30^, ^31^, ^32^, ^33^, ^34^, ^35^, ^36^, ^37^, ^38^ Resource use and costs were estimated separately for each health state, SAE, and drug acquisition related to dapagliflozin (10 mg) and SoC. Costs were estimated in DKK, converted to EUR at an exchange range of 7.46, and adjusted to price year 2024.^39^ The annually cost of dapagliflozin in Denmark were estimated to €769.

**Table 2:** Inputs Weighted Based on Hemodialysis and Peritoneal Dialysis.^a^

Parameter	Mean (SE)	Source
Costs		
Treatment		
Dapagliflozin	€769	^21^
SoC	€13	^21^
CKD management		
CKD G1	€1,389	Assumption
CKD G2	€1,389	Assumption
CKD G3a	€1,389	^24^
CKD G3b	€1,565	^24^
CKD G4	€2,043	^24^
CKD G5, pre-RRT	€3,379	^24^
Dialysis	€50,073	23, 24, 25
Transplant initial^b^	€59,767	^23^^,^^25^, ^26^, ^27^
- Transplant	€11,505	^23^^,^^25^^,^^27^
Events		
HHF	€5,240	^25^
Volume depletion	€21	^22^
Major hypoglycemic event	€176	^28^
Diabetic ketoacidosis	€3,366	^25^
Fracture	€5,596	^25^
Amputation	€11,257	^25^
Utilities		
Health state utilities		
CKD G1	0.85 (0.09)	^29^
CKD G2	0.85 (0.09)	^29^
CKD G3a	0.80 (0.08)	^29^
CKD G3b	0.80 (0.08)	^29^
CKD G4	0.74 (0.07)	^29^
CKD G5, pre-RRT	0.73 (0.07)	^29^
Dialysis^c^	0.49 (0.05)	^22^^,^^30^
Transplant	0.71 (0.07)	^31^
Events-related disutility		
HHF	0.09 (0.01)	^32^
Volume depletion	0.05 (0.01)	^33^^,^^34^
Major hypoglycemic event	0.01 (0.00)	^35^^,^^36^
Diabetic ketoacidosis	0.01 (0.01)	^37^
Fracture	0.15 (0.03)	^33^^,^^34^
Amputation	0.28 (0.06)	^35^^,^^38^

Abbreviations: CKD, chronic kidney disease; HHF, hospitalization for heart failure; MI, myocardial infarction; RRT, renal replacement therapy; SoC, standard of care; T2D, type 2 diabetes.

a Costs are reported annually.

b Weighted based on the proportion with normal versus complicated treatment courses and the proportion receiving a kidney from a living donor versus a deceased donor.^23^

c Weighted based on hemodialysis and peritoneal dialysis.^23^

### Utilities

Utilities and disutilities were measured by EuroQoL-5 Dimension 3 Levels (EQ-5D-3L) data (Table 2). Utilities were applied for each health state and adjusted according to age-specific utilities from the general Danish population.^40^ For each SAE, a specific disutility was applied.

### Total population and subgroup analyses

Cost-effectiveness was evaluated for the total CKD population and stratified across subgroups relevant for assessing the SGLT2i recommendations in Danish CKD guidelines. Hence, the groups evaluated include the following:

• The total CKD population.
• Subgroups stratified on UACR level and T2D status:
  o High UACR (≥200 mg/g) with T2D.
  o High UACR (≥200 mg/g) without T2D.
  o Low UACR (<200 mg/g) with T2D.
  o Low UACR (<200 mg/g) without T2D.
• Subgroups stratified on UACR level and history of HF:
  o High UACR (≥200 mg/g) with prior HF.
  o High UACR (≥200 mg/g) without prior HF.
  o Low UACR (<200 mg/g) with prior HF.
  o Low UACR (<200 mg/g) without prior HF.
• Subgroups stratified on UACR level and age, eGFR, or cardiovascular disease (CVD) (Table S3).

### Model validation

The validity of the model was confirmed by running the model for subgroups corresponding to the trial participants in the DAPA-CKD, DECLARE-TIMI 58, and EMPA-KIDNEY trials with similar time horizons reflecting the published follow-up results.^7^^,^^8^^,^^16^

### Sensitivity analysis

Probabilistic sensitivity analyses (PSAs) were made for all subgroups to evaluate the robustness of the ICERs. Standard error (SE) was assumed to be 30% of the mean cost values and 10% for all remaining values unless literature SE were available. The PSA results were visualized by a cost-effectiveness scatterplot with 500 iterations for each subgroup. NMB were considered for varying time horizons for the broad population and each subgroup to evaluate the evolution of the cost-effectiveness of dapagliflozin (Figs 1 and 2).

**Figure 1:**
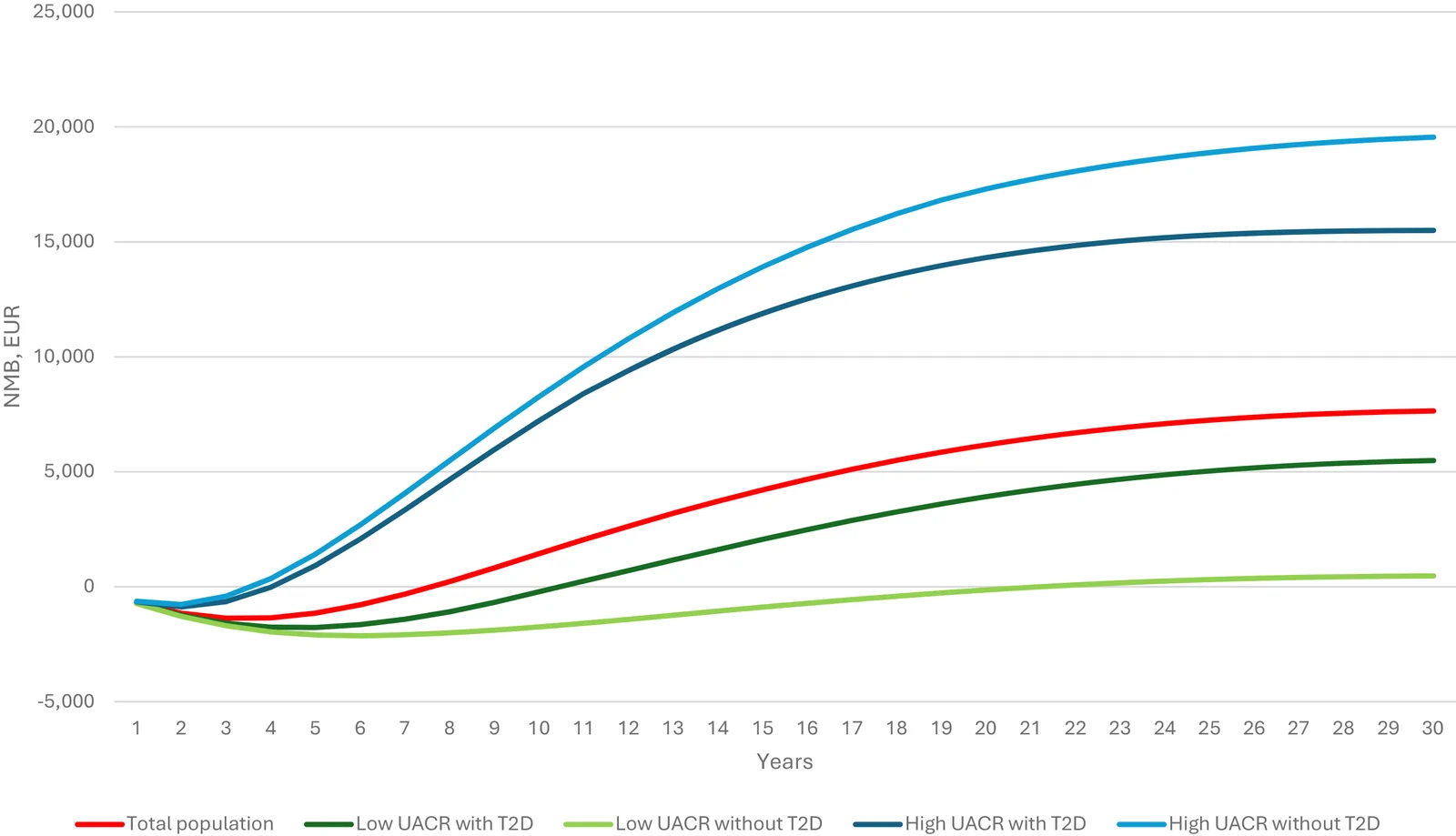
Development of NMB over time by subgroup on UACR level and T2D status. Abbreviations: NMB, net monetary benefit; T2D, type 2 diabetes; UACR, urinary albumin-creatine ratio.

**Figure 2:**
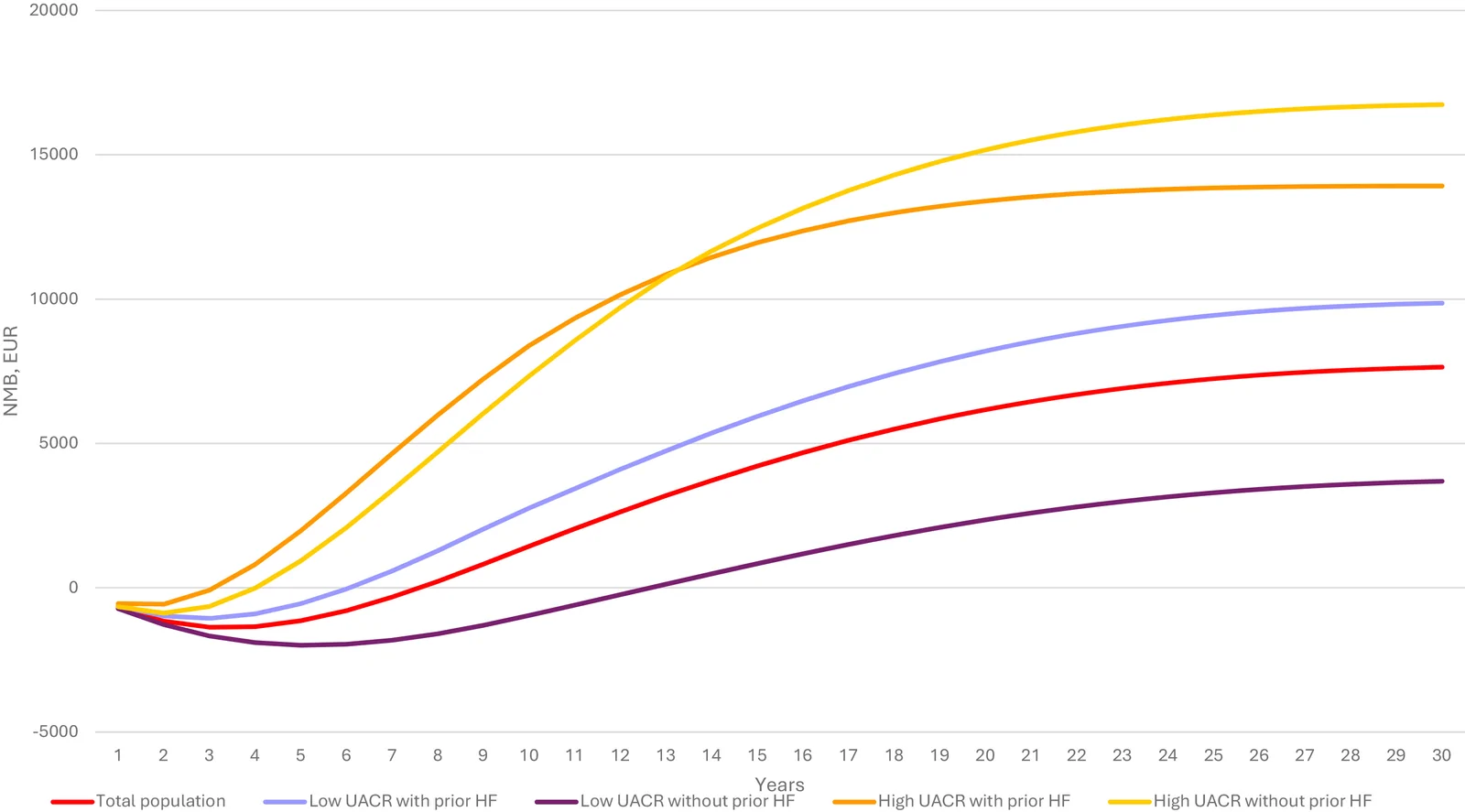
Development of NMB over time by subgroup on UACR level and history HF. Abbreviations: HF, heart failure; NMB, net monetary benefit; UACR, urinary albumin-creatine ratio.

Uncertainties identified in collaboration with clinical experts were investigated through sensitivity analyses. Given that the model predicts outcomes for the total population assuming that 25% have high UACR, additional analyses were made assuming a proportion of 15% with high UACR to encounter that a greater proportion of high risk patients with higher albuminuria were likely to be recruited into clinical trials that in the general population.^6^ Additionally, an analysis was performed to evaluate the impact of a lower CKD transition rate of 0.9 in individuals without T2D compared with those with T2D. Further sensitivity analyses included addition of monitoring costs, adjustments of CKD stage G1 and G2 costs, adjustments of dapagliflozin costs, additional dialysis costs, no discontinuation after dialysis, treatment stopped after 4 years, adjusted baseline population with myocardial infarction (MI), patients exiting the model on RRT, and 0% discounting (Table S3). Additional subgroup analyses included stratification by UACR levels alone and in combination with age (< or > 65 years), eGFR (< or > 45 mL/min/1.73 m^2^), and history of CVD. Furthermore, the impact of the history of HF was considered on the overall population (Table S3). A tornado diagram of deterministic one-way sensitivity analysis is also shown in Figure S2.

### Ethics

According to Danish law, approval from the National Committee on Health Research Ethics was not necessary (§14 stk. 2). Informed consent was waived because of deidentified information.

## Results

Characteristics of the CKD population used to model cost-effectiveness, and the relevant subgroups are included in Tables 1, S1, and S2. In the total model CKD population, mean age was 64 years with 33.3% being female, 73% of White race, and 74.5% having T2D. Most patients were CKD stage 3 (58%) with 22% being stage 3a. Among patients without T2D, 30% had glomerulonephritis as the underlying kidney disease.

The total population analysis resulted in an ICER of €7,499/QALY (ΔCost = €3,441 and ΔQALY = 0.46) well below a WTP threshold of €24,158/QALY and a NMB of €7,643. Sensitivity analyses of the total population showed that ICERs remained below the WTP threshold (Tables 3 and S3). The average QALY gain was 0.55, and LY gain was 0.46. Analyses exploring the developments of the NMB over time demonstrated that dapagliflozin was cost-effective in the total population after approximately 8 years (Figs 1 and 2).

For all subgroups, the ICER fell below the WTP threshold (Table 3). The model indicates that subgroups with high UACR were particularly cost-effective. However, longer time is required for cost-effectiveness to be achieved for patients with low UACR and without T2D or history of HF. For patients with low UACR without T2D time to cost-effectiveness is more than 20 years (Figs 1 and 2). The PSA yielded consistent findings across almost all subgroups (Figs 3 and 4). However, simulations of the subgroup with low UACR without T2D showed a higher degree of uncertainty with respect to cost-effectiveness, with ICERs fluctuating above and below the WTP threshold. Simulations of subgroup analyses by HF history showed limited variation across iterations, primarily because of model assumptions that treatment effects are driven by T2D and CKD status.

**Table 3:** Base Case, Subgroup, and Scenario Analyses Results

	Dapagliflozin + SoC			SoC			ICER	Incremental NMB
	Costs	QALY	LY	Costs	QALY	LY		
Total population	€50,862	9.67	12.38	€47,421	9.21	11.83	€7,499	€7,643
Subgroup analyses								
Low UACR with T2D	€37,510	10.18	12.80	€32,451	9.74	12.27	€11,589	€5,488
Low UACR without T2D^a^	€45,775	9.60	12.41	€43,333	9.48	12.30	€20,243	€472
High UACR with T2D	€86,834	7.81	10.70	€87,227	7.18	9.92	Dominant	€15,500
High UACR without T2D	€100,059	9.06	12.26	€102,482	8.35	11.42	Dominant	€19,553
Low UACR with prior HF	€30,247	7.89	9.86	€25,071	7.27	9.09	€8,316	€9,861
Low UACR without prior HF^b^	€38,132	10.37	13.03	€33,359	10.02	12.62	€13,623	€3,692
High UACR with prior HF	€66,369	6.21	8.42	€65,201	5.59	7.63	€1,870	€13,920
High UACR without prior HF	€93,683	8.40	11.46	€94,994	7.76	10.68	Dominant	€16,739
Sensitivity analyses, total population								
15% with high UACR	€45,518	9.87	12.54	€41,491	9.43	12.02	€9,280	€6,456
CKD transition HR non-T2D: 0.9	€50,570	9.68	12.39	€47,050	9.23	11.84	€7,729	€7,482

Abbreviations: CKD, chronic kidney disease; HF; heart failure; HR, hazard ratio; LY, life years; SoC; standard of care; T2D, type 2 diabetes; UACR, urinary albumin-creatine ratio.

a SGLT2i is not recommended for this group unless they also have a history of HF.

b SGLT2i is not recommended for this group unless they also have T2D and UACR >30 mg/g and eGFR<60 mL/min/1.73 m^2^.

**Figure 3:**
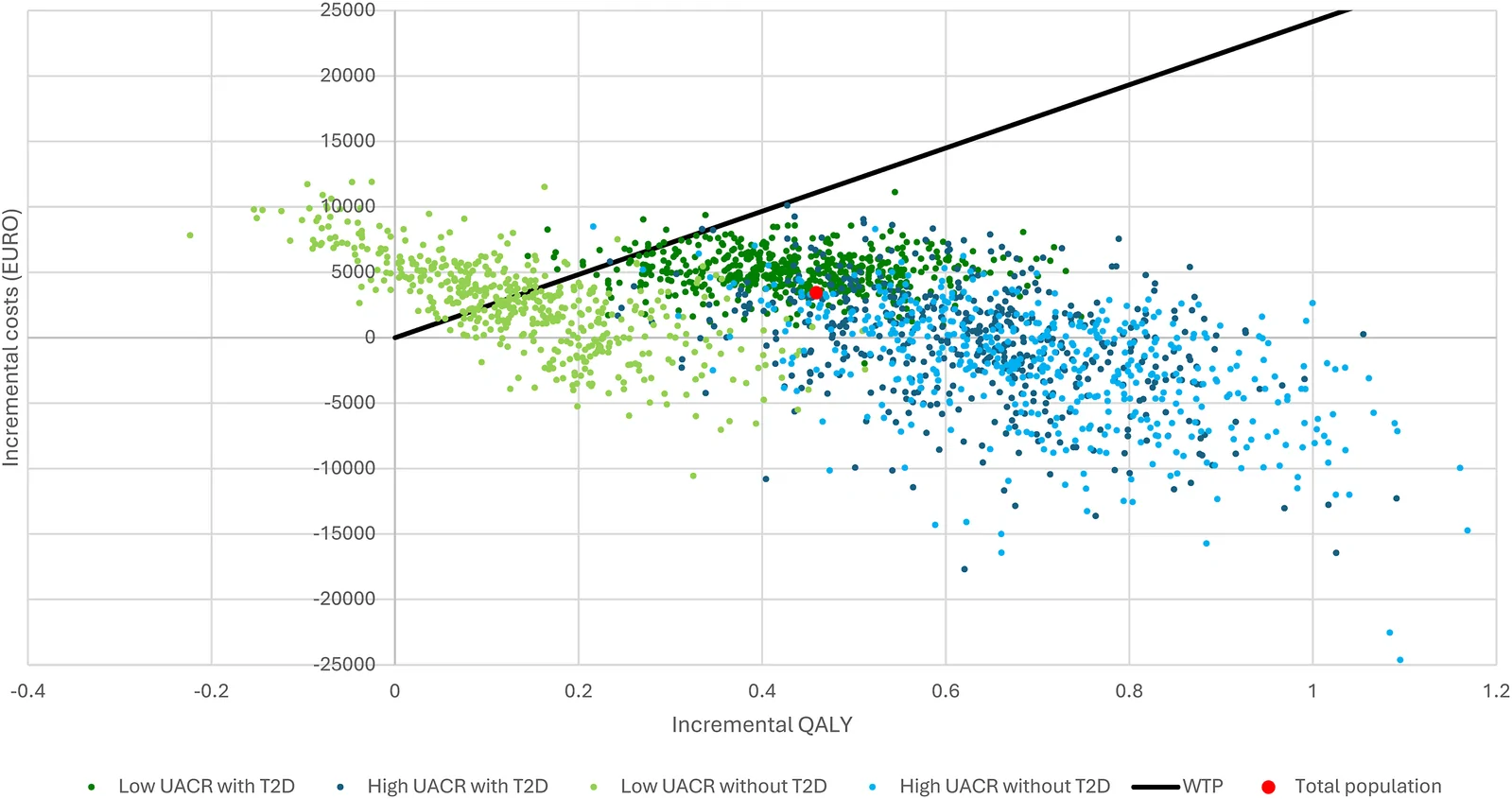
Probabilistic sensitivity analysis by subgroup on UACR level and T2D status.^a^ Abbreviations: QALY, quality-adjusted life years; T2D, type 2 diabetes; UACR, urinary albumin-creatine ratio; WTP: willingness-to-pay. ^a^Iterations under the WTP line are considered cost-effective, whereas iterations above the line are not considered cost-effective.

**Figure 4:**
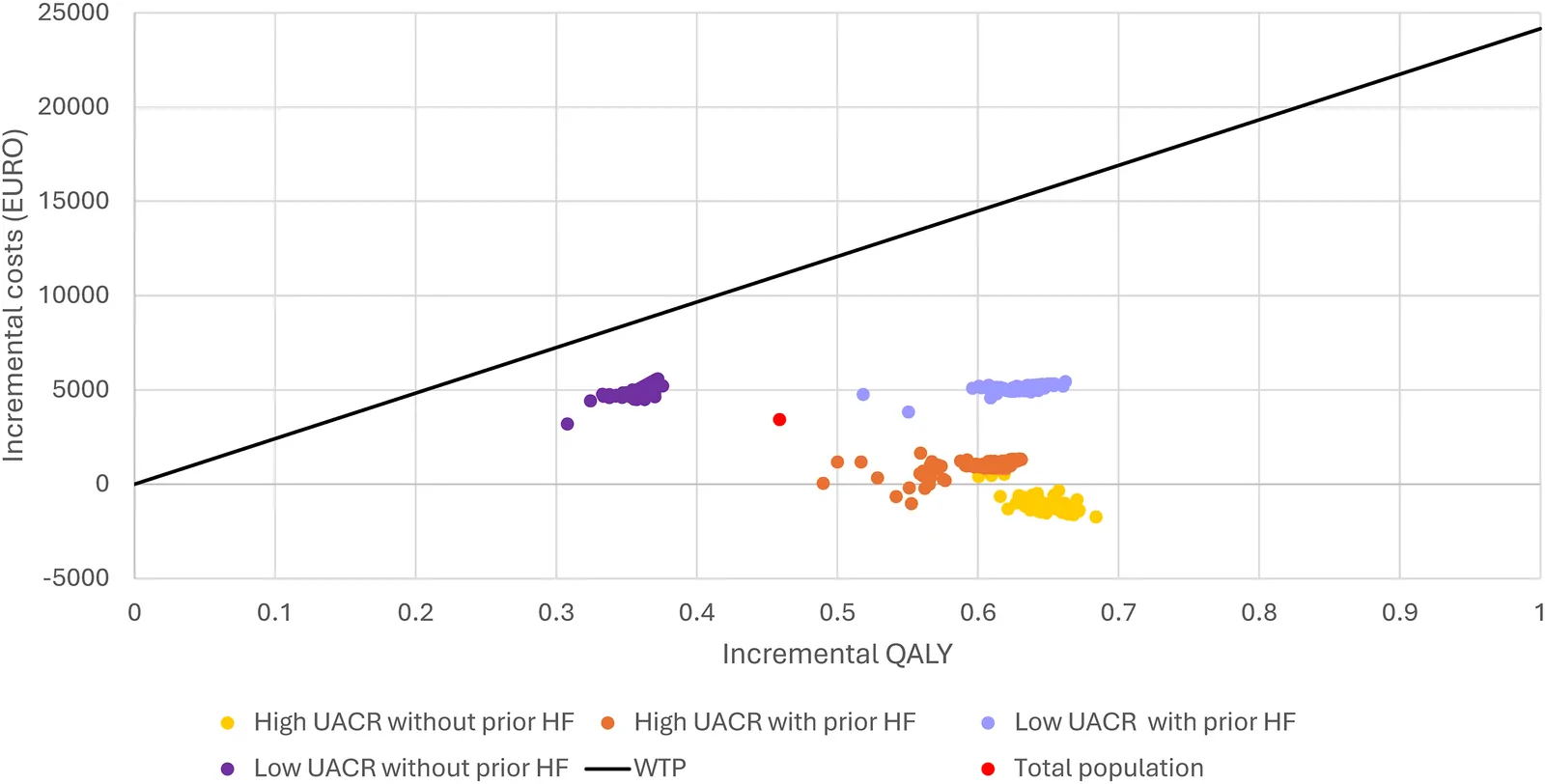
Probabilistic sensitivity analysis by subgroup on UACR level and history HF.^a^ Abbreviations: HF, heart failure; QALY, quality-adjusted life years; UACR, urinary albumin-creatine ratio; WTP: willingness-to-pay. ^a^Iterations under the WTP line are considered cost-effective, whereas iterations above the line are not considered cost-effective.

## Discussion

This study is the first to investigate the cost-effectiveness of treatment in accordance with Danish guidelines. Furthermore, it is the first cost-effectiveness study to incorporate history of HF and detailed UACR subgroup classifications. Based on the model CKD population dapagliflozin treatment is cost-effective compared with SoC in all subgroups recommended SGLT2i in the current Danish guidelines assuming a WTP of €24,158/QALY.

The results in this study support the conclusions from a previously published systematic review on cost-effectiveness of dapagliflozin for patients with CKD irrespective of T2D status.^41^ Additionally, dapagliflozin has shown cost-effectiveness regardless of eGFR, UACR, and T2D status in UK, Spain, Italy and Japan.^15^ However, our base-case estimate of €7,499 per QALY gained is at the lower end of previously published European analyses, likely reflecting the use of real-world baseline characteristics from all identified CKD patients at the hospital level in Denmark, as well as the inclusion of a comprehensive set of cardiovascular events, thereby capturing additional health gains and cost offsets.

The results support the potential value of dapagliflozin in improving health outcomes for CKD patients, while also reducing the economic burden on the health care system. Furthermore, the subgroup analyses highlight the importance of UACR level rather than the history of HF to identify patients for which dapagliflozin is cost-effective, indicating UACR is more important for assessment of cost-effectiveness of SGLT2i in people with CKD. In contrast to eGFR, which is a commonly use marker to identify CKD in Denmark, only about half of incident CKD patients had at least 1 UACR measurement.^42^ Thus, more consistent and frequent UACR monitoring should allow better earlier identification of patients in which SGLT2i are cost-effective.^13^ The result of the present study demonstrates greater cost-effectiveness in patients with high UACR being cost-effective after 4 to 5 years. In contrast, the economic advantage in patients with low albuminuria may take longer to materialize requiring a longer predicted survival to achieve cost-effectiveness. These results suggest that clinical practice should prioritize SGLT2i in CKD patients with elevated albuminuria, particularly those with T2D or HF.^13^ Although generally well tolerated, potential adverse events should be considered in patient selection.

Several limitations should be considered when interpreting these results. Firstly, the study population from the DECLARE-TIMI 58 study was selected based on the presence of multiple risk factors for atherosclerotic cardiovascular disease, which may limit the generalizability of the results to other CKD populations. Second, the median follow-up period in the DAPA-CKD and DECLARE-TIMI 58 trials was 2.4 and 4.2 years respectively, adding uncertainty to any extrapolation beyond this. However, the early demonstration of cost-effectiveness, particularly within 4 to 5 years for subgroups with high UACR, suggest early economic benefit within the study follow-up time. Third, the cost-effectiveness of SGLT2i in the subgroup characterized by low UACR without T2D is inherently subject to uncertainty because of the reliance on simulated patient data. In line with this, the results in the present study were stratified by subgroups to gain a more detailed understanding of the impact of this subgroup. Fourth, the baseline characteristics in the study are based on data from the DAPA-CKD and DECLARE-TIMI 58 trials which do not mirror the Danish CKD population. The CKD patients in the model population were on average approximately 10 years younger than of Danish national register based CKD-cohorts with a greater proportion of males, and fewer patients in CKD 3a.^6^^,^^13^^,^^14^^,^^43^ Furthermore, the model population likely had more patients with type 2 diabetes, fewer patients of White race and more patients with glomerulonephritis when compared Danish CKD patients, as well as a greater than expected proportion of patients with a UACR >30 mg/g in the low UACR subgroup without T2D. Yet, the burden of cardiovascular comorbid conditions, including ischemic heart disease, stroke, and heart failure, was comparable with other Danish CKD cohorts supporting the validity of cardiovascular risk estimates despite age differences. In addition, the sensitivity analyses demonstrated consistent cost-effectiveness results, although it cannot be excluded that differences not accounted for in the sensitivity analyses may affect cost-effectiveness estimates. Fifth, the cost data applied in the analysis is subject to uncertainty because of limited available data from a Danish perspective. Particularly costs related to CKD stages G1 and G2 are subject to uncertainty. Implementation in clinical practice may also entail additional costs not captured in this analysis, including those related to increased UACR testing, patient identification, and treatment initiation. These may affect short-term costs but are unlikely to change the overall cost-effectiveness conclusions. Sensitivity analyses of treatment costs showed no significant impact on the results. Sixth, the assumed WTP threshold does not reflect an official Danish standard for primary care, affecting the interpretation of cost-effectiveness. However, a different, potentially higher Danish-specific threshold has been estimated for the Danish Medicines Council’s prioritization of hospital medicine, and using a higher threshold value would lead to an even more favorable evaluation of SGLT2i.^29^

In conclusion, the results suggest that the current Danish guidelines for treatment with SGLT2i is cost-effective with respect to dapagliflozin at a WTP of approximately €24,000/QALY. The findings also demonstrate that SGLT2i is most cost-effective in CKD patients with high UACR particularly in combination with T2D or history of HF.

## Footnotes

Figure S1. Markov model schematic diagram. Figure S2. Tornado diagram. Table S1. Baseline Characteristics of the Subgroups Related to UACR Levels and T2D Status. Table S2. Baseline Characteristics Divided into Subgroups Related to UACR Levels and History of HF. Table S3. Additional Subgroup and Sensitivity Analyses Over a Lifetime (30 Years) Horizon.
