# Supplementary material for: Cost-Effectiveness of Dapagliflozin in the Treatment of Patients With Chronic Kidney Disease in Denmark
Source: Kidney Med. 2026 Aug 12;8(10):101497. doi: 10.1016/j.xkme.2026.101497 (PMC13634386; doi:10.1016/j.xkme.2026.101497)
Supplement: Supplementary file 1 [file mmc1.pdf]

Supplementary Material

Figure S1. Markov model schematic diagram.

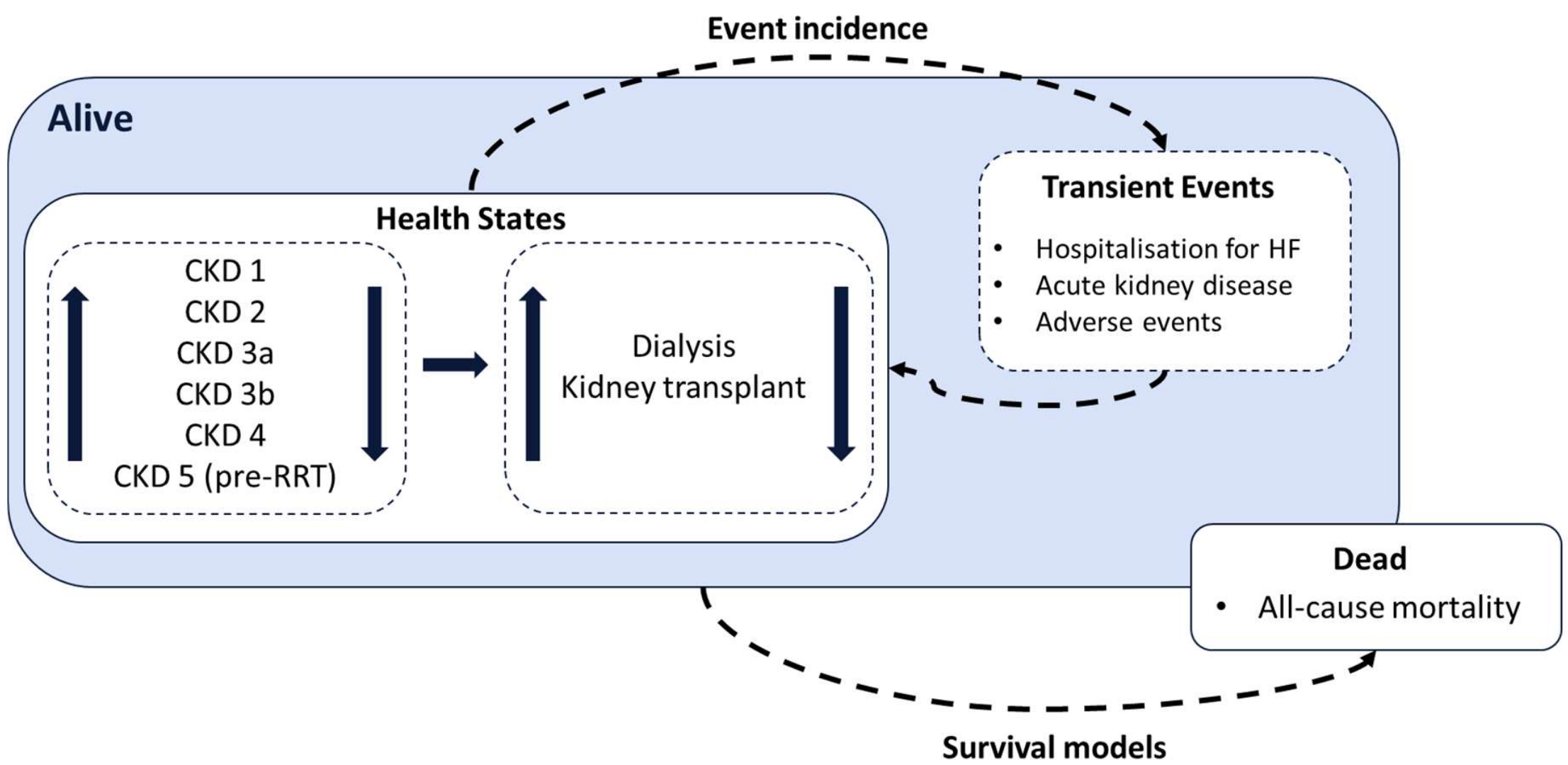

Abbreviations: CKD: Chronic kidney disease; HF: Heart failure; RRT: renal replacement therapy.

- Progression of CKD is modelled using a Markov structure with states defined by CKD stage and RRT (dialysis or transplantation), each assigned a state-specific utility.
- Hospitalisation for HF and acute kidney injury are included as temporary events lasting one cycle, during which additional costs and utility losses apply.
- Transition probabilities and event risks are estimated from patient-level data from the DAPA-CKD trial using multinomial and time-to-event analyses.
- Patients on dapagliflozin have a per-cycle probability of discontinuation; after discontinuation, event risks are assumed equivalent to placebo.
- The figure S1 and description are derived from a previously published cost-effectiveness model [1,2].

Table S1. Baseline characteristics of the subgroups related to UACR levels and T2D status.

	High UACR		Low UACR	
	**With T2D**	**Without T2D**	**With T2D**	**Without T2D**
**Demographic characteristics:**				
Age, years	64.1 (0.13)	56.3 (0.39)	64.6 (0.11)	66.8 (2.78)
Female	31.8 % (0.01)	33.9 % (0.01)	33.7 % (0.007)	33.3 % (0.10)
BMI, kg/m^2	31.2 (0.10)	27.9 (0.15)	32.4 (0.091)	26.6 (0.77)
Race				
- White	61.0 % (0.01)	53.8 % (0.01)	77.8 % (0.01)	41.7 % (0.10)
- Black or African American	4.4 % (0.00)	3.9 % (0.01)	3.6 % (0.00)	4.2 % (0.04)
- Asian*	26.1 (0.03)	38.1 (0.04)	15.3 (0.02)	45.8 (0.05)
- Other	8.5 % (0.00)	4.2 % (0.01)	3.4 % (0.00)	8.3 % (0.06)
Smoker	37.9 % (0.01)	42.3 % (0.01)	14. 7% (0.01)	33.3 % (0.10)
**Clinical characteristics:**				
CKD G1	14.5 % (0.01)	0.0 % (0.00)	37.9 % (0.01)	0.0 % (0.00)
CKD G2	24.2 % (0.01)	7.7 % (0.01)	37.6 % (0.01)	0.0 % (0.00)
CKD G3a	23.9 % (0.01)	29.0 % (0.01)	20.5 % (0.01)	45.8 % (0.10)
CKD G3b	28.3 % (0.01)	47.2 % (0.01)	3.6 % (0.00)	45.8 % (0.10)
CKD G4	9.2 % (0.00)	16.1 % (0.01)	0.3 % (0.00)	8.3 % (0.06)
CKD G5 (pre-RRT)	0.0 % (0.00)	0.0 % (0.00)	0.0 % (0.00)	0.0 % (0.00)
Dialysis	0.0 % (0.00)	0.0 % (0.00)	0.0 % (0.00)	0.0 % (0.00)
Transplant	0.0 % (0.00)	0.0 % (0.00)	0.0 % (0.00)	0.0 % (0.00)
UACR: 30-300 mg/g	14.3 % (0.00)	8.2 % (0.01)	82.8 % (0.006)	100.0 % (0.00)
UACR: >=300 mg/g	85.8 % (0.00)	91.9 % (0.01)	0.0 % (0.00)	0.0 % (0.00)
T2D	100.0 % (0.00)	0.0 % (0.00)	100.0 % (0.00)	0.0 % (0.00)
Glomerulonephritis	3.4 % (0.00)	43.1 % (0.01)	1.2 % (0.012)	25.0 % (0.09)
ACE	37.3 % (0.01)	32.9 % (0.01)	49.1 % (0.01)	37.5 % (0.10)
ARB	57.9 % (0.01)	65.5 % (0.01)	36.1 % (0.01)	58.3 % (0.10)
MRA	5.7 % (0.00)	4.1 % (0.01)	5.0 % (0.00)	4.2 % (0.04)
Diuretic	47.7 % (0.01)	28.4 % (0.01)	45.0 % (0.01)	20.8 % (0.09)
Potassium, mEq/L	4.6 (0.01)	4.6 (0.01)	4.5 (0.007	4.5 (0.11)
Systolic blood pressure, mmHg	140.3 (0.26)	132.7 (0.45)	135.9 (0.24)	130.0 (2.77)
Haemoglobin, g/dL	13.0 (0.03)	13.3 (0.05)	13.8 (0.02)	13.4 (0.35)
**Medical history:**				
Prior HF	12.8 % (0.01)	7.6 % (0.01)	11.9 % (0.01)	12.5 % (0.07)
Prior MI	22.6 % (0.01)	5.0 % (0.01)	15.4 % (0.01)	12.5 % (0.07)
Prior stroke	8.4 % (0.00)	4.8 % (0.01)	7.4 % (0.00)	8.3 % (0.06)

Mean unless otherwise indicated (SE).
*SE 10% of mean value.
Abbreviations: ACE; Angiotensin-converting enzyme, ARBs; Angiotensin receptor blockers, BMI; Body mass index, HF; Heart failure, MI; Myocardial infarction, RRT; Renal replacement therapy, T2D; Type 2 diabetes, UACR; Urinary albumin-to-creatine ratio.

Table S2. Baseline characteristics divided into subgroups related to UACR levels and history of HF.

	High UACR		Low UACR	
	**Prior HF**	**No prior HF**	**Prior HF**	**No prior HF**
**Demographic characteristics:**				
Age, years	64.8 (0.36)	61.9 (0.16)	65.6 (0.33)	64.5 (0.11)
Female	33.2 % (0.02)	31.9 % (0.01)	33.6 % (0.02)	33.7 % (0.01)
BMI, kg/m^2	32.6 (0.26)	30.1 (0.09)	34.0 (0.27)	32.2 (0.10)
Race				
- White	81.4 % (0.02)	56.4 % (0.01)	91.6 % (0.01)	75.7 % (0.01)
- Black or African American	5.7 % (0.01)	4.1 % (0.00)	2.8 % (0.01)	3.7 % (0.00)
- Asian*	9.7 (0.01)	31.5 (0.03)	3.4 (0.00)	17.1 (0.02)
- Other	3.1 % (0.01)	8.0 % (0.00)	2.2 % (0.01)	3.5 % (0.00)
Smoker	35.3 % (0.02)	39.4 % (0.01)	13.3 % (0.02)	15.0 % (0.01)
**Clinical characteristics:**				
CKD G1	10.0 % (0.01)	11.1 % (0.00)	29.0 % (0.02)	38.9 % (0.01)
CKD G2	22.5 % (0.02)	20.0 % (0.01)	36.1 % (0.02)	37.6 % (0.01)
CKD G3a	26.1 % (0.02)	25.0 % (0.01)	29.0 % (0.02)	19.5 % (0.01)
CKD G3b	30.7 % (0.02)	33.0 % (0.01)	4.9 % (0.01)	3.7 % (0.00)
CKD G4	10.8 % (0.01)	10.8 % (0.00)	0.9 % (0.00)	0.3 % (0.00)
CKD G5 (pre-RRT)	0.0 % (0.00)	0.0 % (0.00)	0.2 % (0.00)	0.1 % (0.00)
Dialysis	0.0 % (0.00)	0.0 % (0.00)	0.0 % (0.00)	0.0 % (0.00)
Transplant	0.0 % (0.00)	0.0 % (0.00)	0.0 % (0.00)	0.0 % (0.00)
UACR: 30-300 mg/g	12.3 % (0.01)	12.9 % (0.01)	75.9 % (0.02)	83.8 % (0.01)
UACR: >=300 mg/g	87.7 % (0.01)	87.1 % (0.01)	0.0 % (0.00)	0.0 % (0.00)
T2D	84.4 % (0.01)	75.1 % (0.01)	79.6 % (0.00)	73.1 % (0.00)
Glomerulonephritis	5.3 % (0.01)	17.7 % (0.01)	0.0 % (0.00)	7.6 % (0.03)
ACE	46.0 % (0.02)	35.0 % (0.01)	55.7 % (0.02)	48.2 % (0.01)
ARB	49.1 % (0.02)	61.1 % (0.01)	31.2 % (0.02)	36.9 % (0.01)
MRA	17.5 % (0.02)	3.7 % (0.00)	21.7 % (0.02)	2.7 % (0.00)
Diuretic	65.4 % (0.02)	40.1 % (0.01)	72.0 % (0.02)	41.2 % (0.01)
Potassium, mEq/L	4.6 (0.02)	4.6 (0.01)	4.5 (0.02)	4.5 (0.01)
Systolic blood pressure, mmHg	139.7 (0.62)	138.3 (0.24)	134.2 (0.71)	136.1 (0.25)
Haemoglobin, g/dL	13.3 (0.07)	13.1 (0.03)	13.7 (0.07)	13.8 (0.02)
**Medical history:**				
Prior HF	100.0 % (0.00)	0.0 % (0.00)	100.0 % (0.00)	0.0 % (0.00)
Prior MI	30.7 % (0.02)	10.6 % (0.00)	44.9 % (0.02)	19.5 % (0.01)
Prior stroke	11.8 % (0.01)	7.0 % (0.00)	10.8 % (0.01)	6.9 % (0.00)

Mean unless otherwise indicated (SE).
*SE 10% of mean value.
Abbreviations: ACE; Angiotensin-converting enzyme, ARBs; Angiotensin receptor blockers, BMI; Body mass index, HF; Heart failure, MI; Myocardial infarction, RRT; Renal replacement therapy, T2D; Type 2 diabetes, UACR; Urinary albumin-to-creatine ratio.

Table S3. Additional subgroup and sensitivity analyses over a lifetime (30 years) horizon.

	Dapagliflozin + SoC			SoC			ICER	Incremental NMB
	Costs	QALY	LY	Costs	QALY	LY		
Subgroup analyses								
**Low UACR**	€37,500	10.16	12.78	€32,595	9.77	12.31	€12,370	€4,675
**High UACR**	€90,948	8.19	11.17	€91,900	7.54	10.38	Dominant	€16,547
**Low UACR, <65 years**	€40,937	11.50	14.21	€35,782	11.07	13.72	€12,076	€5,157
**Low UACR, >65 years**	€32,892	8.58	11.07	€28,353	8.24	10.65	€13,494	€3,587
**High UACR, <65 years**	€99,973	8.92	12.11	€101,488	8.24	11.30	Dominant	€17,963
**High UACR, >65 years**	€74,125	6.72	9.53	€75,200	6.22	8.89	Dominant	€13,173
**Low UACR, eGFR <45**	€48,745	9.05	11.74	€45,704	8.62	11.22	€7,026	€7,415
**Low UACR, eGFR >45**	€36,993	10.26	12.83	€32,002	9.82	12.36	€12,638	€4,549
**High UACR, eGFR <45**	€92,938	8.18	11.22	€95,495	7.47	10.36	Dominant	€19,508
**High UACR, eGFR >45**	€89,439	8.19	11.14	€89,145	7.59	10.39	€487	€14,289
**Low UACR, prior CVD**	€35,056	9.48	11.87	€29,753	8.94	11.20	€9,786	€7,788
**Low UACR, no prior CVD**	€38,267	10.38	13.07	€33,653	10.07	12.71	€14,987	€2,823
**High UACR, prior CVD**	€79,405	7.27	9.95	€79,379	6.63	9.15	€41	€15,305
**High UACR, no prior CVD**	€96,683	8.65	11.78	€98,200	8.01	11.00	Dominant	€16,739
**Overall with prior HF**	€39,277	7.47	9.50	€35,103	6.85	8.72	€6,700	€10,876
Sensitivity analyses								
**Addition of monitoring costs**	€50,880	9.67	12.38	€47,421	9.21	11.83	€7,539	€7,625
**CKD G1 and G2 costs 0%**	€41,689	9.67	12.38	€38,900	9.21	11.83	€6,076	€8,296
**CKD G1 and G2 costs 200%**	€60,036	9.67	12.38	€55,942	9.21	11.83	€8,923	€6,990
**Dapagliflozin costs 50%**	€47,995	9.67	12.38	€47,421	9.21	11.83	€1,251	€10,510
**Dapagliflozin costs 10%**	€45,700	9.67	12.38	€47,421	9.21	11.83	Dominant	€12,805
**Dialysis cost +20%**	€54,899	9.67	12.38	€52,020	9.21	11.83	€6,277	€8,204

**Stop treatment after 4 years**	€49,050	9.41	12.06	€47,421	9.21	11.83	€8,200	€3,170
**No discontinuation after dialysis**	€52,284	9.68	12.41	€47,421	9.21	11.83	€10,268	€6,579
**Adjusted MI in baseline characteristics. High UACR subgroup: 23% [3], Low UACR subgroup: 10%**	€50,853	9.71	12.44	€47,434	9.27	11.90	€7,729	€7,268
**Patients exit model upon RRT**	€26,294	9.35	11.78	€19,586	8.85	11.15	€13,413	€5,374
**Discounting 0%**	€74,156	13.17	17.08	€69,733	12.44	16.18	€6,091	€13,115

Abbreviations: CKD; Chronic kidney disease, CVD; Cardiovascular disease, eGFR; Estimated glomerular filtration rate, HF; Heart failure, ICER; Incremental cost-effectiveness ratio, LY; Life years, MI; Myocardial infarction, NMB; Net monetary benefit, QALY; Quality-adjusted life years, RRT; Renal replacement therapy, SoC; Standard of care, UACR; Urinary albumin-to-creatine ratio.

Figure S2. Tornado diagram

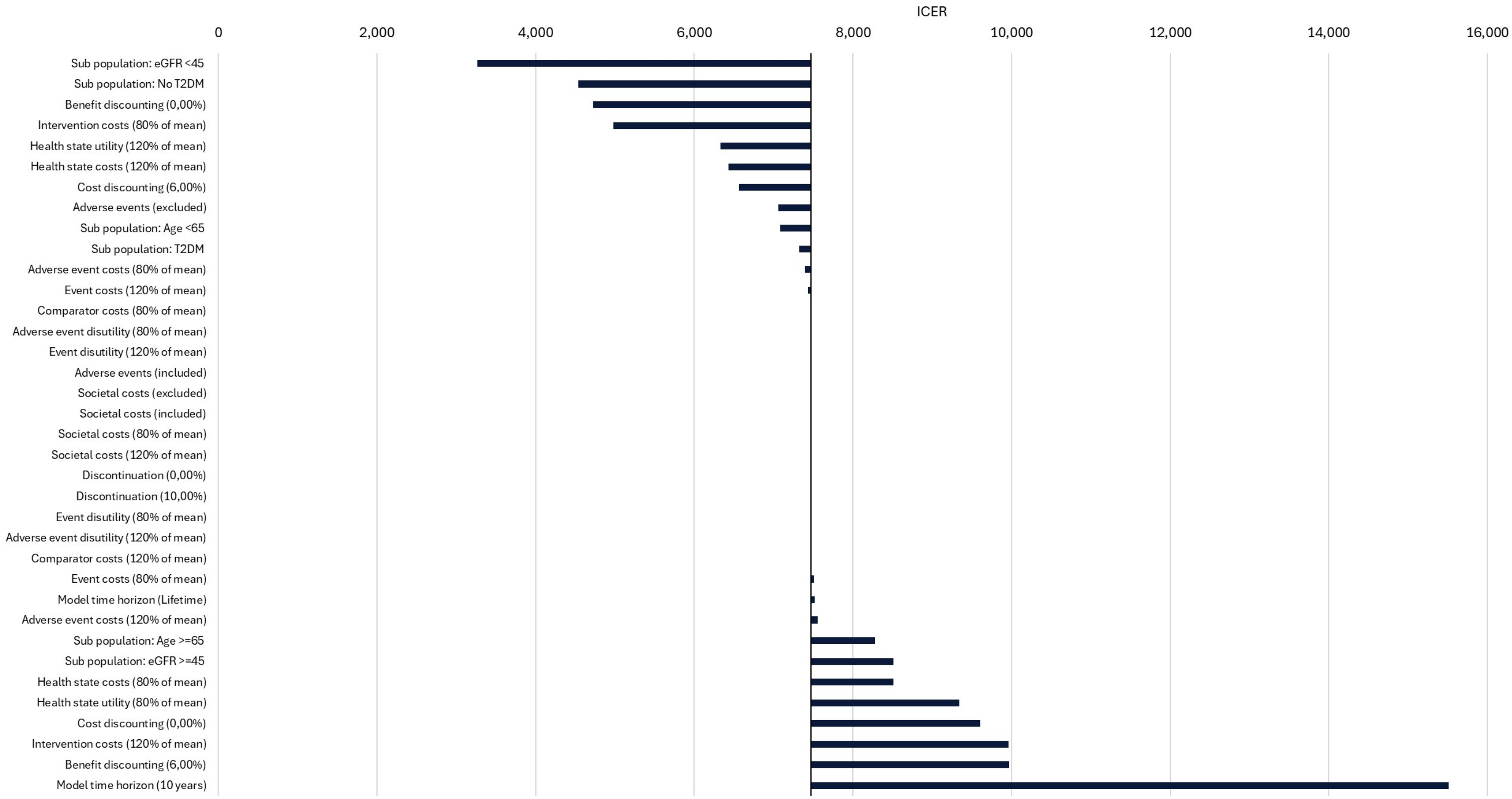

Supplementary References

1. McEwan P, Darlington O, Miller R, et al. Cost-Effectiveness of Dapagliflozin as a Treatment for Chronic Kidney Disease: A Health-Economic Analysis of DAPA-CKD. *CJASN*. 2022;17(12):1730-1741. doi:10.2215/CJN.03790322

2. McEwan P, Davis JA, Gabb PD, et al. Dapagliflozin in chronic kidney disease: cost-effectiveness beyond the DAPA-CKD trial. *Clinical Kidney Journal*. 2024;17(2):sfae025. doi:10.1093/ckj/sfae025

3. Perkovic V, Tuttle KR, Rossing P, et al. Effects of Semaglutide on Chronic Kidney Disease in Patients with Type 2 Diabetes. *N Engl J Med*. 2024;391(2):109-121. doi:10.1056/NEJMoa2403347
